# Repair of an Isolated Sternal Head Tear of the Myotendinous Junction of the Pectoralis Major Using Knotless Suture Anchors: Case Report and Literature Review

**DOI:** 10.7759/cureus.65806

**Published:** 2024-07-30

**Authors:** Khaled K AlAbbasi, Mustafa AlRawi, Amr Elmaraghy

**Affiliations:** 1 Department of Orthopedic Surgery, King Fahad Medical City, Riyadh, SAU; 2 Department of Orthopaedic Surgery, University of Toronto, Toronto, CAN

**Keywords:** pectoralis major tendon rupture, pectoralis major rupture, trauma of pectoralis majors, orthopedic surgery shoulder and elbow and upper extremity, upper extremity reconstruction, upper extremity trauma, upper-extremity

## Abstract

Although considered a relatively uncommon sports injury, publications on pectoralis major (PM) injuries have increased in the last couple of decades. Knowledge of the complex anatomy of the PM muscle is important in diagnosing, understanding the complexity of the injury, and determining the suitable modality of management of these injuries. Despite the increase in publications, there is no consensus on the superiority of any proposed surgical management.

We present a case of a recreational body builder who presented to our clinics with a rare pattern of isolated musculotendinous junction of the lower fibers of the PM muscle and proposed a new technique of surgical management of such injuries using knotless suture anchors and running locked suture pattern in different directions. We then conducted a comprehensive review of literature of these injuries and presented a review on the pathophysiology, the various patterns of these injuries, and the available described modalities of surgical management.

Understanding the complex anatomy of the PM, the various pattens of injury, and the aid of an MRI read by an expert musculoskeletal radiologist is crucial before managing these injuries. We believe that acute surgical repair of musculotendinous junction injuries using running Krackow/Brunnell locked configuration and the use of knotless suture and anchors will provide adequate and practicable surgical repair of these injuries.

## Introduction

The reported incidence of pectoralis major (PM) injures is less the 60 in 100,000 per year in professional athletics [1]. The incidence has significantly increased within the last two to three decades due to the increase in participation in recreational sports activities, especially weightlifting, as well as a greater awareness of these injuries. These injuries are more prevalent in athletic males in the age range of 20 to 30 years old [1-7]. Despite this increase in incidence, PM muscle injuries remain relatively rare compared to other sports injuries.

Knowledge of the specific complex anatomic characteristics of the PM muscle is critical in understanding the pathology of the injury. The PM muscle originates in two heads: the smaller clavicular head and the larger sternal head. The muscle fibers of the clavicular head originate from the medial aspect of the clavicle and travel laterally in a semi-horizontal course towards its insertion. In contrast, the sternal head muscle fibers, which account for more than 80% of the PM muscle bulk, originate from the second to the sixth rib, the associated costa-cartilage, and the external oblique muscle. The upper sternal head fibers also course distally and caudally below the clavicular head and they both contribute to the anterior tendon layer. The lower sternal head fibers course supero-laterally and pass deep to contribute to the posterior tendon layer. The lower sternal head is also the main component of the anterior axillary fold [4,8-10]. The anterior and posterior tendon layers and their contributing muscle fibers converge together to form the combined PM tendon, inserting on the lateral aspect of the intertubercular sulcus. A deeper look into the anatomy of the PM tendon reveals a 5 cm flat tendon and bilaminate structure where the muscle fibers of the clavicular head and the upper muscle fibers of the sternal head contribute to the anterior lamina of the tendon, while the posterior lamina is formed by the lower sternal head muscle fibers [4,9].

Tendon injuries at or near its insertion and the less common myo-tendinous junction injuries occur mostly due to eccentric contraction while the muscle is maximally stretched [2,4]. This usually occurs during the last phase of extension in bench pressing exercising while the arms are in abduction, external rotation and near full extension [2,6,11]. The shorter overall muscle fiber length of the inferior portion of the sternal muscle fibers, which are then forced during this activity over a longer complex changing course coursing superolateral first and then inferolateral towards their insertion helps explains this segment’s tendency to fail first. Further duration and/or magnitude of force can then cause the more caudal portion of the muscle fibers of the sternal head, and lastly the tendon portion formed by the clavicular head to fail in more severe injuries

Multiple methods of acute surgical management of PM muscle injuries have been described in the literature, including direct repair, bone tunneling, standard anchor sutures, cortical button suture anchors, and open reduction and internal fixation in the rare cases of bony avulsion [3,12-18]. No available comparative studies proved superiority of any of these methods. In this study we are reporting a case of an isolated myotendinous rupture of the sternal head of the PM muscle and the surgical technique we used to manage this injury, followed by a literature review of the classification, outcomes, and various surgical techniques described for these injuries. 

## Case presentation

**Figure 1:**
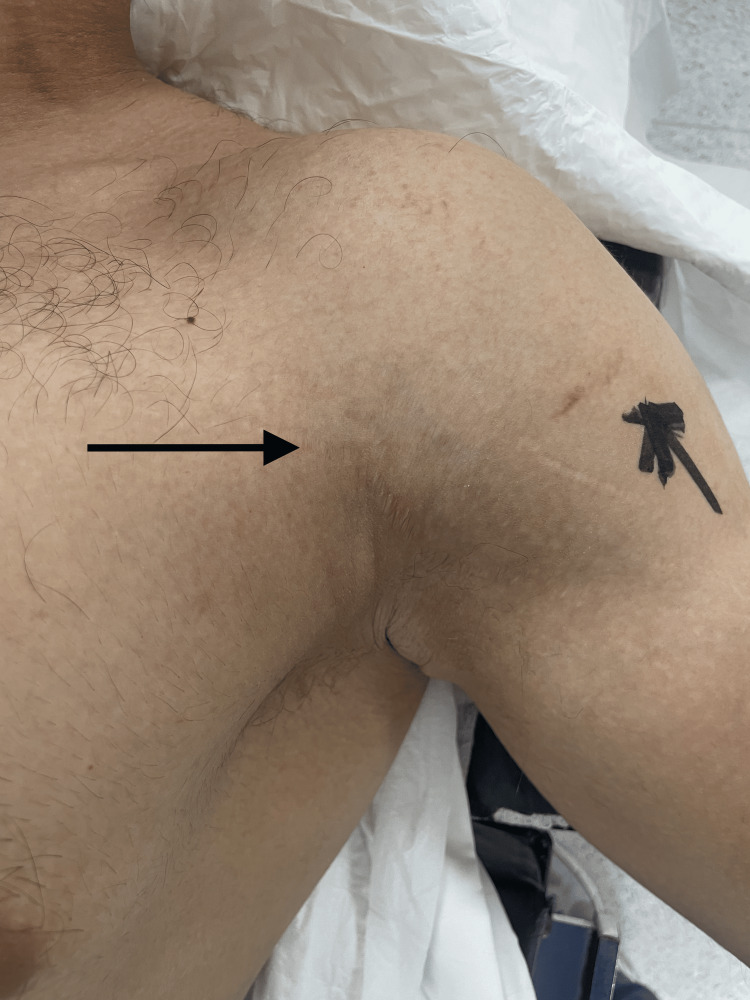
Loss of the axillary fold due to injury of the myotendinous junction of the pectoralis major muscle.

A 26-year-old male patient presented to the upper-limb orthopedic surgery outpatient clinic complaining of pain and bruising in the left upper arm and axilla region. After a detailed history and physical examination, it was found that the patient's history dates to five days prior to presentation to our clinic, when he heard a loud popping sound in his left shoulder region followed by acute severe pain in the left upper arm while doing bench pressing exercises. The patient’s arm gave away and he was unable to continue exercising. The next day he noticed that the swelling and bruising had increased. The patient admitted to a sporadic use of steroids in the past, but denied being currently on any anabolic medications. On physical examination, the patient had bruising in the left upper arm and axilla with an asymmetry of both the anterior axillary fold and the overall PM contour (Figure 1).

**Figure 2:**
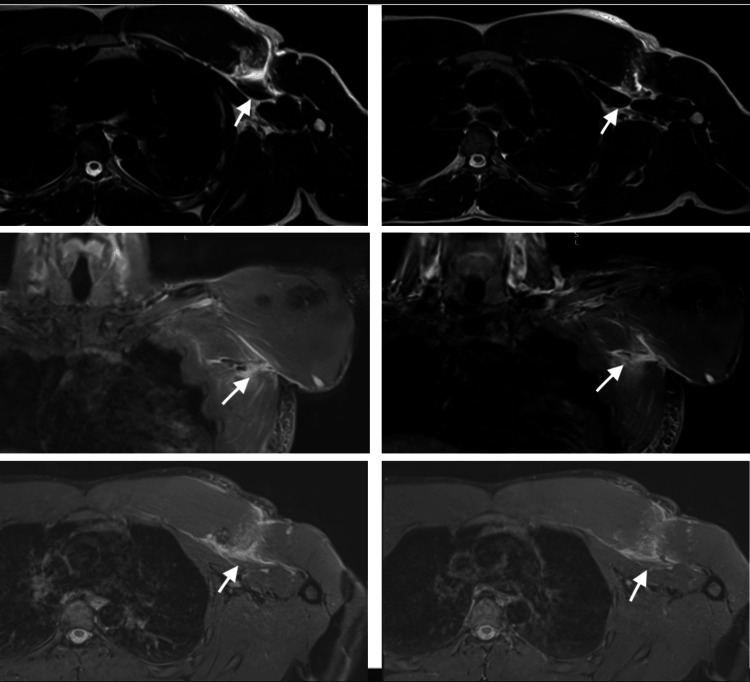
MRI scan of the left chest showing a rupture at the myotendinous junction of the sternal head of pectoralis major with retraction and associated diffuse edema in the involved muscle and large hematoma in the gap between the humerus and the retracted muscle segments. The transverse measurement of the tear gap was 4.5 cm with an intact tendinous bony insertion measured length of 2.5 cm.

Further examination demonstrated a decreased active range of motion (ROM) and weakness, both of which were attributable to pain. An MRI was ordered and reviewed by an expert musculoskeletal radiologist (Figure 2). After discussing the findings and the management options and recognizing his desire to return to recreational weightlifting at the same level as pre-injury, the patient consented for surgical repair of the PM.

Surgical technique

At the time of surgery, it was one week after the injury. The procedure was performed under general anesthesia and in the beach chair position. The injured arm and chest were prepped and draped lateral to the sternum and in a fashion that allowed free movement of the entire limb. The forearm was then connected to a mechanical arm-supporting device. The whole surgical field and the axilla were covered with an Ioban drape (3M, St Paul, MN, USA). 

**Figure 3:**
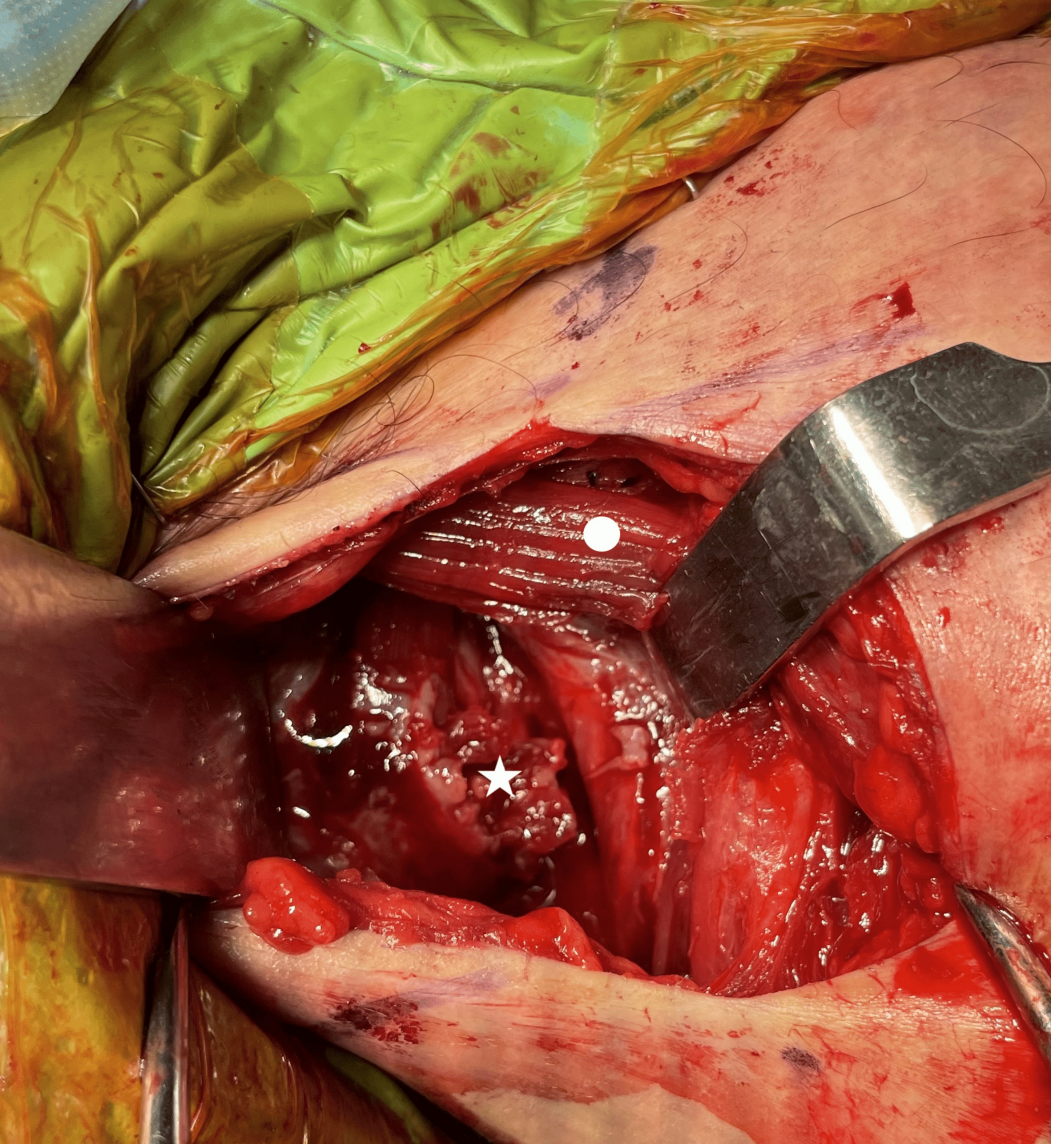
The myotendinous stump (white star) is identified by the medial retraction of the skin and superolateral retraction of the deltoid and the clavicular head muscle belly (white circle).

A 5 cm vertical incision was made starting 1 cm distal to the coracoid process and directed towards the axilla. A more vertical incision, rather than the classical oblique delto-pectoral approach skin incision, was chosen to provide a better exposure medially to identify the retracted PM musculotendinous junction. After incising the skin, the subcutaneous layer superficial to the brachial and delto-pectoral fascia was raised as a full-thickness flap, both medially and laterally. The delto-pectoral interval was utilized distally only to identify the site of insertion of the PM tendon. In our case, since the disruption was at the musculo-tendinous junction, 2 to 2.5 cm of the tendon was observed to have remained intact on the lateral aspect of the intertubercular sulcus. The myotendinous stump of the sternal head was identified by superior-lateral retraction of the deltoid muscle and intact clavicular head muscle fibers and gentle blunt dissection infero-medially in the subcutaneous tissue (Figure 3).

**Figure 4:**
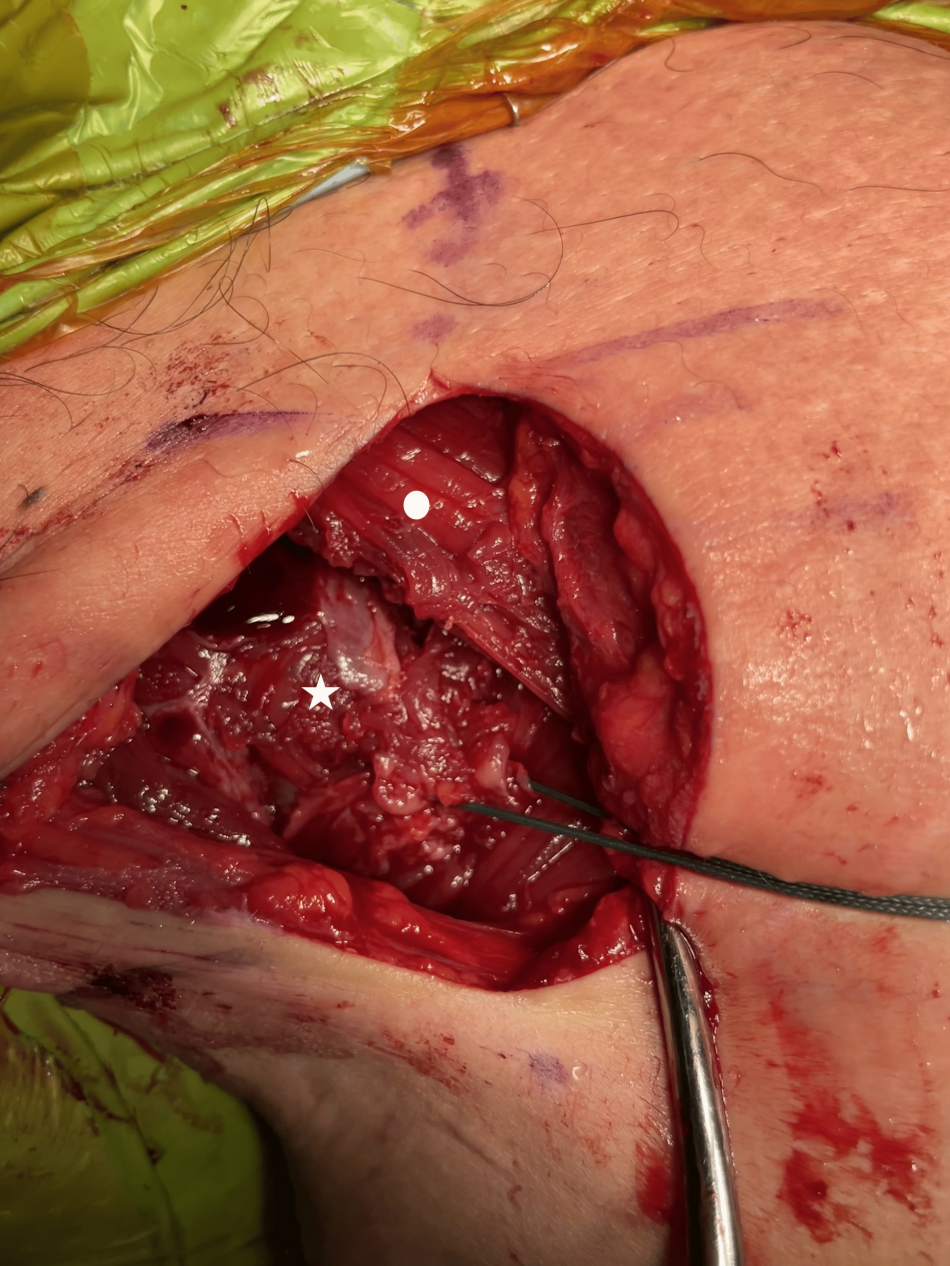
A retracting suture was placed in the myotendinous stump (white star) to pull it laterally. The clavicular head is labeled by a white star.

After identifying the stump, the muscle belly was mobilized both superficially and deep to allow maximum excursion of the muscle fibers laterally. A temporary traction suture was placed into the stump to allow tensioning of the retracted stump (Figure 4).

**Figure 5:**
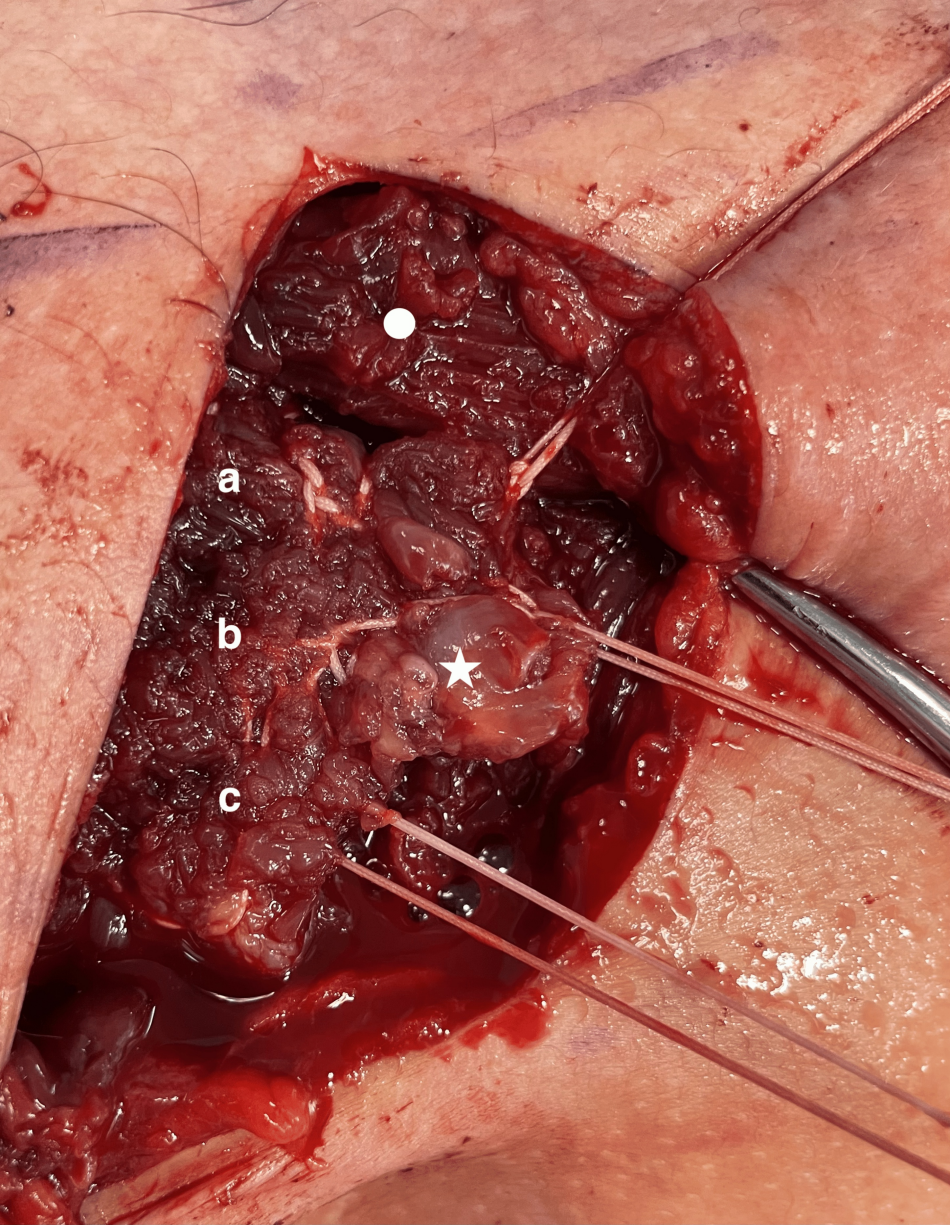
Three FiberWire sutures were placed in the upper (a), middle (b) and lower parts (c) of the myotendinous stump (white star) using running Krackow technique and ensuring to run the suture 5 cm into the muscle belly before returning to the edge of the stump. The clavicular head is labeled by a white star.

A #5 FiberWire suture (Arthrex, Naples, FL, USA) was passed through the upper part of the stump using a Krackow technique. The suture was started in the upper end of the stump and progressed in a locking fashion for around 5 cm medial to the stump and then back in the same fashion ensuring to include the deep fascia with the muscle fibers to avoid muscle necrosis. This was repeated in the middle and lower part of the stump; taking into consideration the trajectory of the muscle fibers of each part and ensuring always to catch the whole bulk of the muscle fibers with each suture passage while not cutting through previous stitches (Figure 5). 

**Figure 6:**
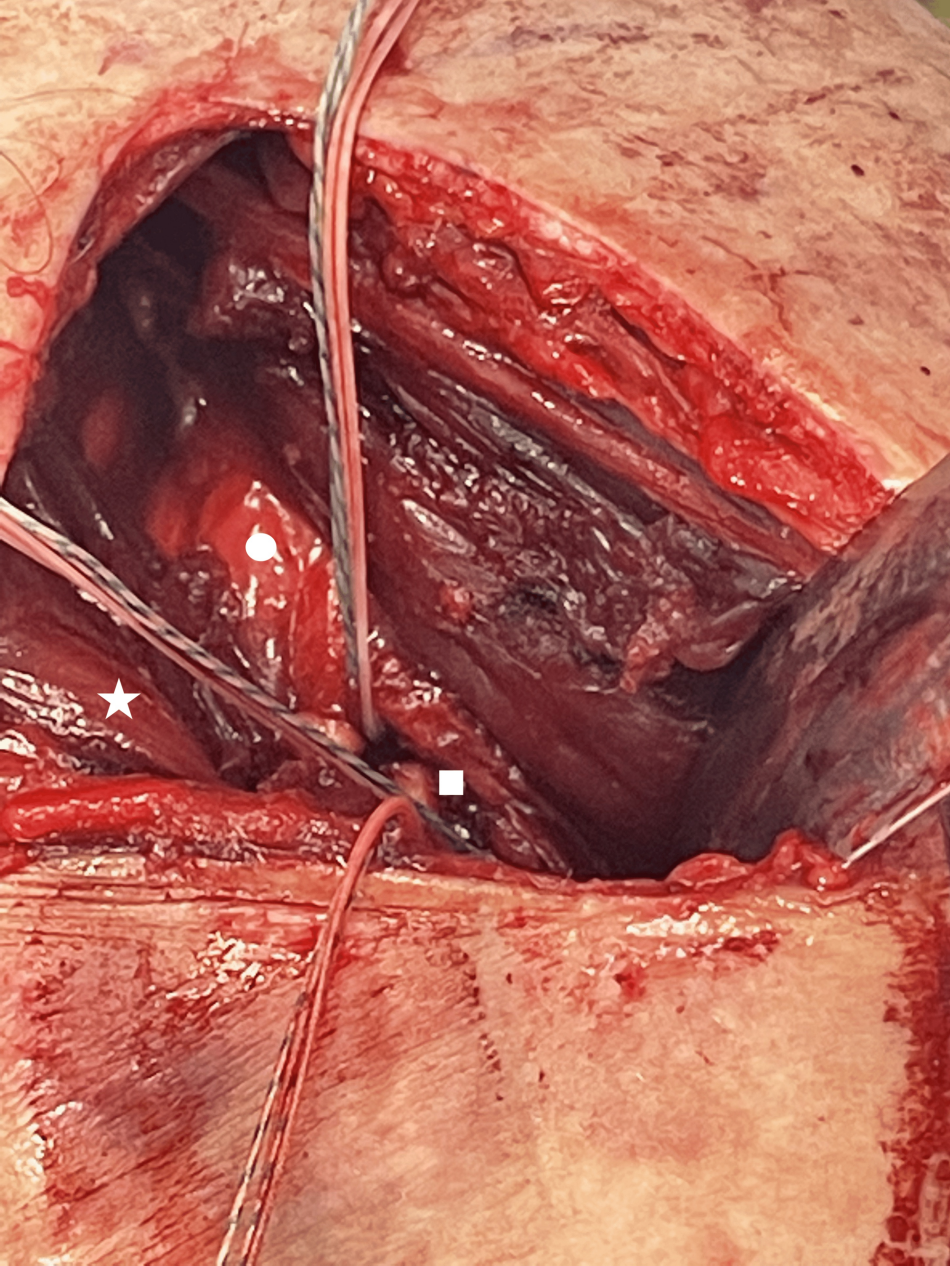
The myotendinous stump (white star) is reattached back to its insertion site (white square) using three 4.5 mm knotless anchor sutures. The anchors are inserted into the bone 1 cm apart, posterior to the remaining intact tendon, directing the upper anchor in a 45-degree angle superiorly, the middle anchor perpendicular to the bone and the lower anchor in a 45-degree angle inferiorly. The stump was then augmented by suturing it to the remaining intact tendon. The clavicular head is labeled by a white star.

To repair the myotendinous stump back to its insertion site, we used three 4.5 mm knotless suture anchors (Pushlock SP; Arthrex). The sutures passed in the upper aspect of the stump were loaded into the uppermost anchor suture, which was inserted in the posterosuperior aspect of the remaining intact tendon and directed superiorly at a 45-degree angle. The middle sutures loaded into the middle suture anchor which was directed horizontally. Finally, the lower sutures were loaded into the lower suture anchor which was directed inferiorly at a 45-degree angle. All suture anchors were inserted immediately posterior to the remaining intact PM tendon with a minimum of 1 cm spacing between each suture anchor (Figure 6).

The suture anchors were inserted and tensioned while the arm was placed in an adducted and internally rotated position. To further augment the repair, we used a # 0 coated Vicryl suture (Ethicon, Cincinnati, OH, USA) to incorporate the stump to the remaining intact sternal head tendon using a locking suture technique, taking care not to damage any of the previously passed FiberWire sutures. At completion of the repair, adequate length-tension and anterior axillary fold contour restoration were evaluated by gentle passive abduction and external rotation of the arm. The wound was irrigated with normal saline and closed in layers. The arm was placed in a sling for protection.

Postoperatively, the patient was kept in a sling for four weeks after surgery. The patient was encouraged to start active elbow flexion and extension on day one. Pendular exercises of the shoulder were initiated at two weeks. Four weeks post-surgery, the patient was started on active and active assisted exercises of the shoulder with caution to avoid external rotation beyond neutral. Isometric strengthening exercises were not started until 10 weeks post-surgery. The patient was allowed to slowly progress his recreational weightlifting activities, with care to employ good form, starting four months of surgery.

## Discussion

**Figure 7:**
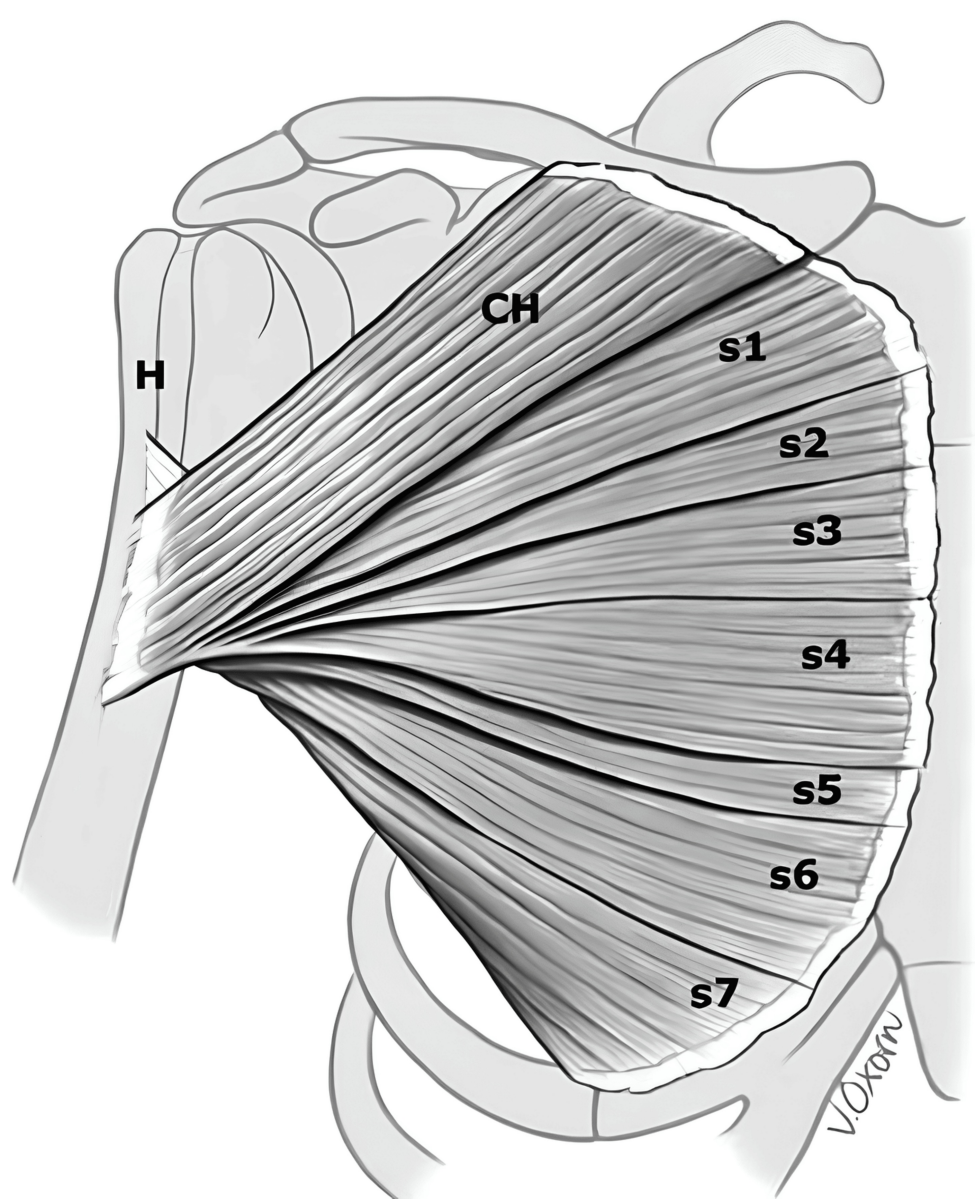
the orientation of the pectoralis major muscle components. The clavicular head and sternal head segments overlap each other in a fanned-out orientation laterally, and medially converge into a combined bilaminar tendon. Figure is taken with permission from: A systematic review and comprehensive classification of pectoralis major tears. [4].

**Figure 8:**
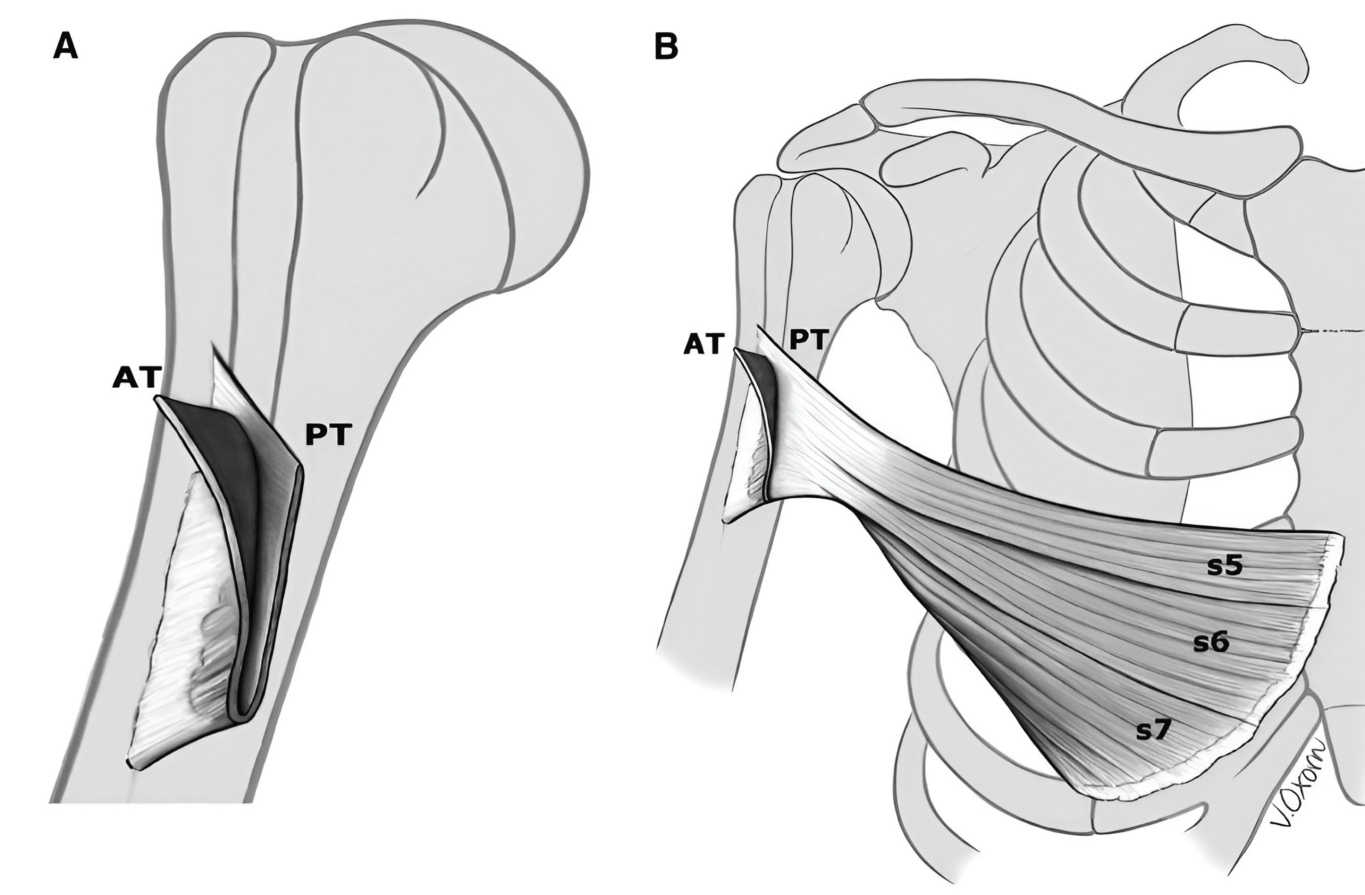
(A) The pectoralis major muscle tendon is a bilaminate structure, connected inferiorly. (B) The anterior tendon layer (AT) is formed by the clavicular head and the upper segments of the sternal head. The posterior tendon layer (PT) is formed by the lower segments of the sternal head muscle fibers. Figure taken with permission from: A systematic review and comprehensive classification of pectoralis major tears. [4].

An indirect mechanism of excessive eccentric contraction of the muscle while the arm is in abduction, external rotation and near full extension, is the major cause of PM injuries, accounting for 83% of all cases [2,6]. These injuries propagate in a cascade starting in the lower part of the sternal head muscle followed by the upper part and finally the clavicular head. The progression of injury in this fashion is due to the shorter length of muscle fibers of the lower aspect of the sternal head compared to the upper part and the acute angle of insertion of these fibers in relation to the direction of action of the muscle compared to the upper part of the sternal head and the clavicular head (Figures 7, 8). In an anatomical study by Wolfe et al., they found a two-time increase in the excursion of the muscle fibers of the lower part of the sternal head muscle while the shoulder was in 30 degrees of extension compared to the upper part and clavicular head where excursion was kept steady throughout the shoulder motion [8].

The first classification used to classify PM muscle injuries was developed by Tietjen in 1980 [12]. This simple classification, which was based on the anatomical site and the extent of the injury, consisted of Type I which included contusion or sprain of the muscle, Type II which included partial injuries, and Type III which included full tears. Type III was further divided into Type IIIA: muscle origin injuries, Type IIIB: muscle belly, Type IIIC: musculotendinous junction, and Type IIID: tendon injuries. This classification was further modified by Bak et al. [2] by adding Type IIIE which included bony avulsion injuries and Type IIIF which included tendon substance injuries. The major drawback of the Tietjen classification and its modification by Bak is its simplicity, as it does not correlate with the complex nature of the PM anatomy and actual injury patterns, so it had minimal input on the management. A better understanding of the anatomy of the PM muscle and the cascade of injuries occurring resulted in the development of a more clinically relevant classification; the Cordasco classification (Table 1), which classified these injuries based on the anatomical location of the injury, but more importantly differentiated between single head and combined head injuries [3].

**Table 1:** Cordasco classification

Cordasco classification [3]		
Type I: contusion or strain		
Type II: tear of an isolated head		
	A: muscle origin	
	B: muscle belly	
	C: myotendinous junction	
	D: tendon	
Type III: tear of both heads		
		A: muscle origin
		B: muscle belly
		C: myotendinous junction
		D: tendon
		E: bony avulsion

This differentiation between single and combined head injuries is quite important because of its clinical importance on the type of surgical repair used. In fact, the increase in publications on PM muscle injuries within the last one to two decades has increased the awareness of the single-head injury group which accounts for 30-50% of PM injuries [1,3,6,7]. Nevertheless, neither the Cardasco classification nor any other study has addressed the prevalence, clinical importance and surgical recommendations for lower sternal head injuries. 

While conservative treatment is reserved for very medial injuries involving exclusively the muscle belly as well as injuries that occur in low-demand elderly patients with multiple medical problems, surgical management is usually the treatment of choice when dealing with active patients with injuries involving the myotendinous junction. Surgical repair has been demonstrated to provide the best chance for a good functional and cosmetic outcome [1,2,4,5,13]. In a meta-analysis by Bak et al. [2] on 112 patients with PM muscle injuries, it was found that the outcome was much better in the group managed surgically compared to those who were treated conservatively (excellent or good: 88% vs 27%). This percentage even increased to 90% vs 17% when patients who were treated initially with conservative management and shifted to delayed surgical management were excluded. Furthermore, patients who had surgical management within eight weeks from injury had better outcomes with better satisfaction. Nevertheless, most studies defined acute injuries as those treated within three to six weeks from injury [4,8,14]. More recent studies have reported over 90% return to pre-injury functional level and 8 to 17% strength deficit when compared to conservative management [3-7,13]. Delaying surgical management beyond six weeks may result in significant muscle retraction and extensive adhesions, thus requiring more complex surgeries with wider approaches, increased operative time, the potential for a higher rate of complications and an increased possibility that tissue grafts reconstruction will be required. Furthermore, delayed surgery can compromise the adequate excursion of the muscle back to its normal insertion without excessive repair site tension, which can lead to higher failure rates and inferior cosmetic outcomes [4,5].

Factors determining the choice of surgical repair method employed include the location of the rupture (myotendinous, intertendinous, insertional site or avulsions), the extent of the injury (single or double head, partial or complete) and the timing of the injury (acute vs chronic) [3,4]. To the best of our knowledge, no previous paper in the literature has clinically compared the different modalities of surgical management of these injuries in order to determine the superiority of any one method of repair. In a biomechanical study by Sheman et al. comparing trans-osseous repair, standard suture anchors, and endosteal cortical button anchors, there was no statistical superiority in any of the biomechanical properties of the three groups, while all constructs were inferior to the biomechanical characteristics of the native PM tendon insertion [19]. With regards to the best suture configuration, the only data available can be found in a biomechanical study by Gregory et al., who reported that using a running locked configuration, i.e Krackow/Brunnell stitch was biomechanically superior to other types of stitch patterns in terms of tissue pull-out strength [20].

## Conclusions

Based on the current available evidence, we propose a new technique for the repair of sternocostal myotendinous junction using running Kruckow/Brunell stitch and knotless anchor sutures and structure-to-structure direct repair. The use of knotless suture anchors has not been described before. We believe that this technique is reproducible, minimizes the risk of failure by both suture pullout and suture breakage, and allows for gradual and better tensioning of the stump back to the tendon footprint. On the contrary, the pull out strength and the best type of anchors to be used should be subjected to further studying. Nevertheless, despite the significant increase in publication on these injuries within the last decade, we still lack clinical studies comparing the different methods of surgical fixation and stitch configuration for different types of PM injuries.
